# Supplementary material for: An embeddable molecular code for Lewis X modification through interaction with fucosyltransferase 9
Source: Commun Biol. 2022 Jul 13;5:676. doi: 10.1038/s42003-022-03616-1 (PMC9279290; doi:10.1038/s42003-022-03616-1)
Supplement: Supplementary file 2 — Supplemental Material [file 42003_2022_3616_MOESM2_ESM.pdf]

Supplementary information

An embeddable molecular code for Lewis X modification through interaction with fucosyltransferase 9

Taiki Saito[1,2], Hirokazu Yagi[1,2], Chu-Wei Kuo[4], Kay-Hooi Khoo[4], and Koichi Kato[1,2,3 *]

[1] Graduate School of Pharmaceutical Sciences, Nagoya City University, 3-1 Tanabe-dori, Mizuho-ku, Nagoya 467-8603, Japan

[2] Institute for Molecular Science, National Institutes of Natural Sciences, 5-1 Higashiyama, Myodaiji-cho, Okazaki, 444-8787, Japan

[3] Exploratory Research Center on Life and Living Systems (ExCELLS), National Institutes of Natural Sciences, 5-1 Higashiyama, Myodaiji-cho, Okazaki, 444-8787, Japan

[4] Institute of Biological Chemistry, Academia Sinica, 128, Academia Road Sec. 2, Nankang, Taipei 115, Taiwan

[*]Addresses for correspondence: Koichi Kato, Ph.D., Exploratory Research Center on Life and Living Systems and Institute for Molecular Science, National Institutes of Natural Sciences, 5-1 Higashiyama, Myodaiji, Okazaki, Aichi 444-8787, Japan, Tel. +81-564-59-5225, Fax: +81-564-59-5224, e-mail: kkatonmr@ims.ac.jp

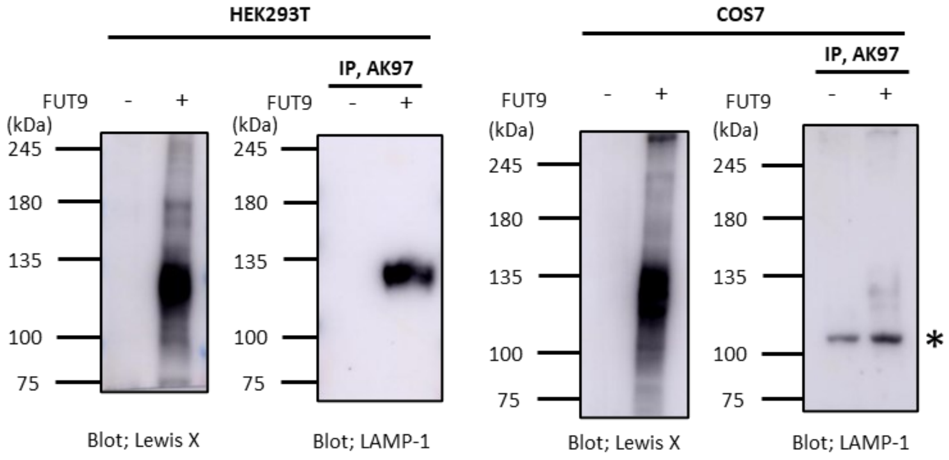

Supplementary Fig. 1. FUT9-dependent Lewis X formation on LAMP-1 in HEK293T and COS7 cells.

Lysates from HEK293T or COS7 cells transfected with or without FUT9 were subjected to immunoblot analysis using the anti-Lewis X antibody, AK97. The cell lysates were also immunoprecipitated with AK97 (IP) and subjected to immunoblot analysis with anti-LAMP-1 antibody. Asterisk indicates a band of unidentified cross-reactive protein.

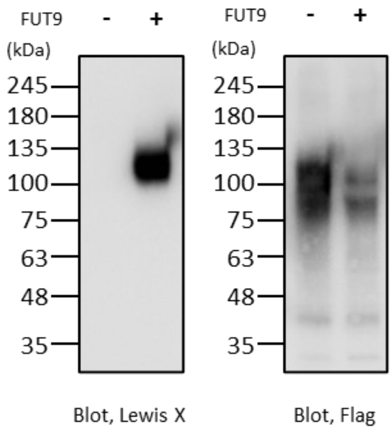

Supplementary Fig. 2. FUT9-dependent Lewis X formation on recombinantly expressed LAMP-1.

The recombinant LAMP-1 glycoproteins with a C-terminal 3xFlag-tag with or without FUT9 overexpression were purified by anti-Flag M2 Affinity Gel, and then subjected to immunoblot analysis using anti-Lewis X, AK97 and anti-Flag antibodies.

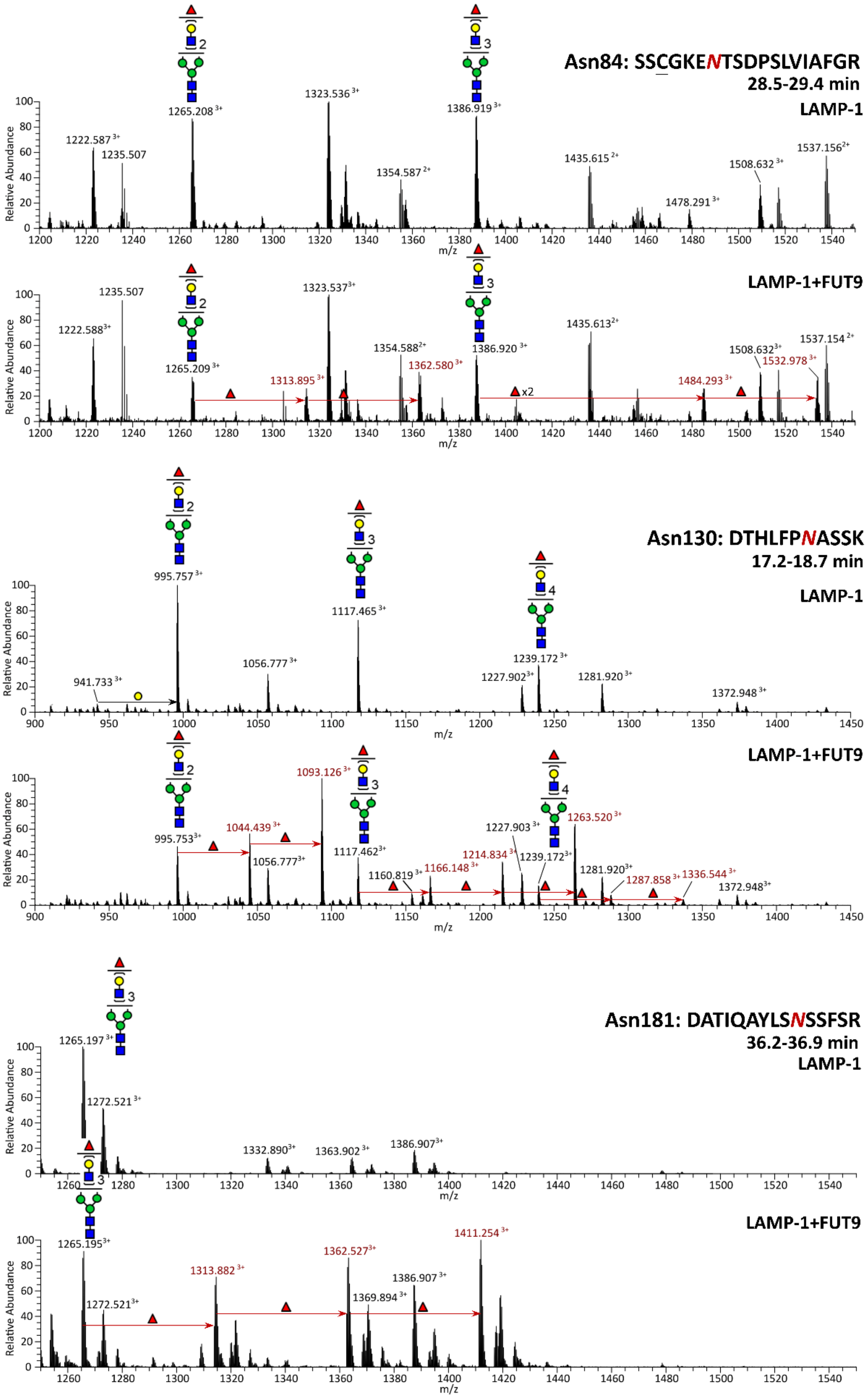
Asn84: SSCGKENTSDPSLVIAFGR
28.5-29.4 min
LAMP-1
LAMP-1+FUT9
Asn130: DTHLFPNASSK
17.2-18.7 min
LAMP-1
LAMP-1+FUT9
Asn181: DATIQAYLSNSSFSR
36.2-36.9 min
LAMP-1
LAMP-1+FUT9
Relative Abundance
m/z
1222.587 3+
1235.507
1265.208 3+
1323.536 3+
1354.587 2+
1386.919 3+
1435.615 2+
1478.291 3+
1508.632 3+
1537.156 2+
1222.588 3+
1265.209 3+
1313.895 3+
1323.537 3+
1354.588 2+
1362.580 3+
1386.920 3+
1435.613 2+
1484.293 3+
1508.632 3+
1532.978 3+
1537.154 2+
x2
941.733 3+
995.757 3+
1056.777 3+
1117.465 3+
1227.902 3+
1239.172 3+
1281.920 3+
1372.948 3+
995.753 3+
1044.439 3+
1056.777 3+
1093.126 3+
1117.462 3+
1160.819 3+
1166.148 3+
1214.834 3+
1227.903 3+
1239.172 3+
1263.520 3+
1281.920 3+
1287.858 3+
1336.544 3+
1372.948 3+
1265.197 3+
1272.521 3+
1332.890 3+
1363.902 3+
1386.907 3+
1265.195 3+
1272.521 3+
1313.882 3+
1362.527 3+
1369.894 3+
1386.907 3+
1411.254 3+

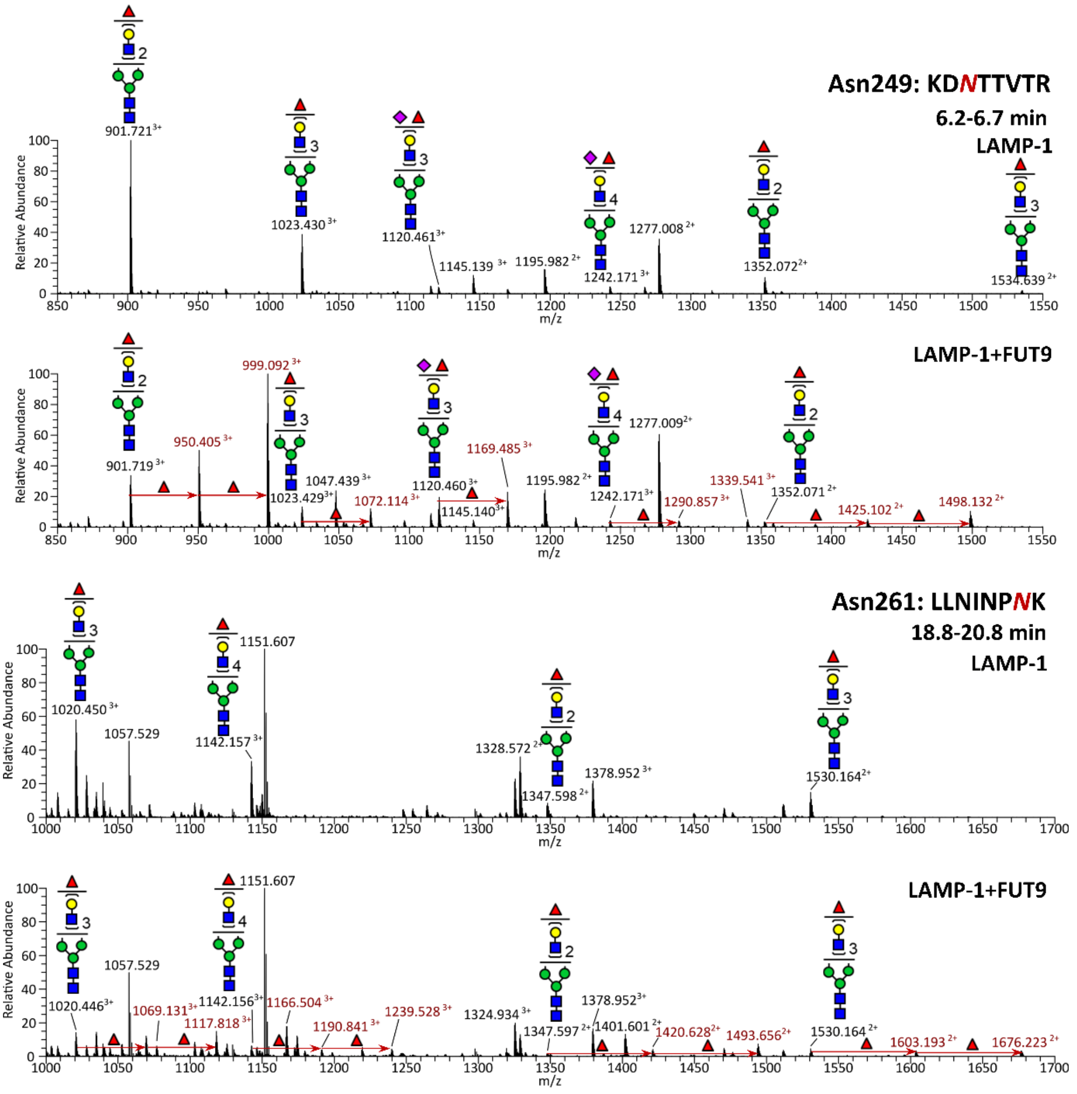

Supplementary Fig. 3. Comparisons of the LC-MS profiles of N-glycosylated peptides from LAMP-1 produced in wild-type and FUT9-expressing CHO-K1 cells.

The MS profiles of tryptic peptides containing Asn84, Asn130, Asn181, Asn249, and Asn261 showed the increment of fucose in FUT9-expressing cells comparing to wild-type. The MS spectra were averaged according to the time ranges labeled in the figures, respectively. The glycan structures were identified by Byonic (Protein Metrics Inc.) and manually assigned using Xcalibur Software.

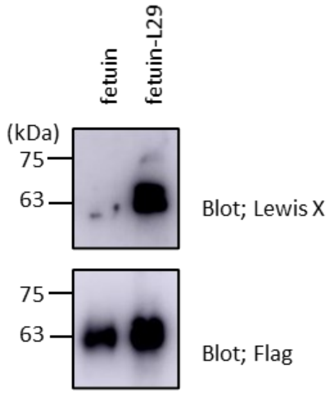

Supplementary Fig. 4. FUT9-dependent Lewis X modification of fetuin dependent upon the L29 sequence.

The recombinant 3xFlag-tagged fetuin glycoproteins with or without a C-terminal L29 sequence tag were subjected to immunoblot analysis using anti-Lewis X, AK97 and anti-Flag antibodies.

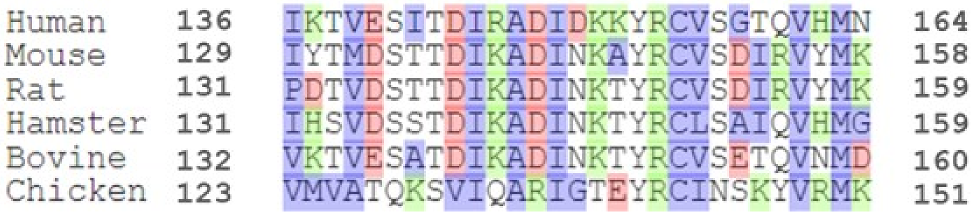

Supplementary Fig. 5. Evolutional conservation of L29 sequence.

Multiple sequence alignment of the consecutive amino acid residues corresponding to the L29 sequence in LAMP-1 of Human (UniProt P11279), Mouse (UniProt P11438), Rat (UniProt P14562), Hamster (UniProt P49129), Bovine (UniProt Q05204), and Chicken (UniProt P05300). Residues highlighted in purple, light green, pink are hydrophobic, positive-charged and negative-charged, respectively.

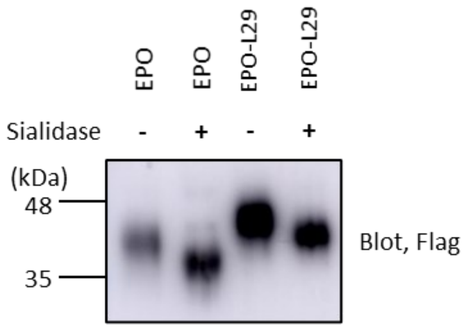

Supplementary Fig. 6. Desialylation of EPO glycoproteins with or without a C-terminal L29 sequence tag.

Full desialylation was confirmed for the recombinant 3×Flag-tagged EPO glycoproteins based on their band sifts. They were subsequently subjected to immunoblot analysis using anti-Flag M2 antibody.

Fig. 1a

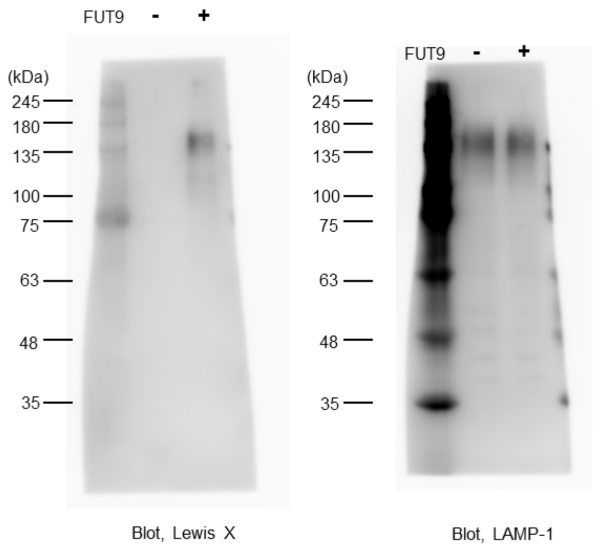

Fig. 1b

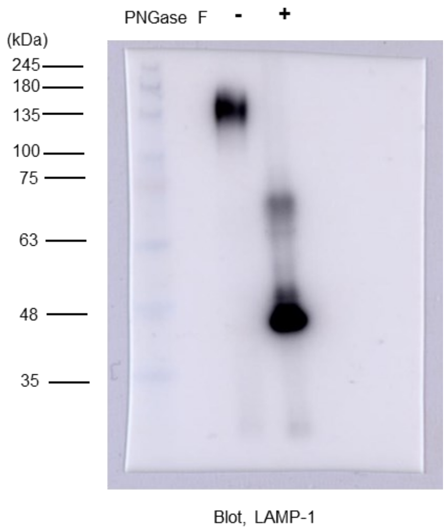

Fig. 1c

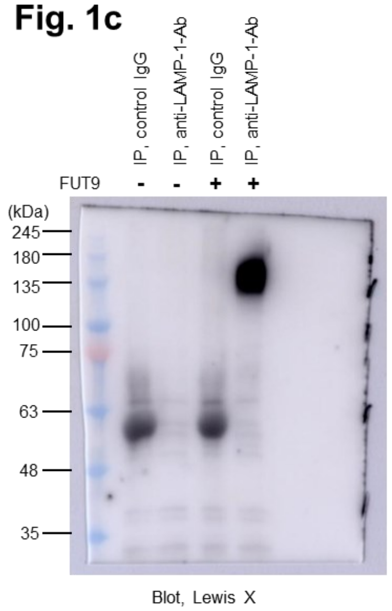

Fig. 1d

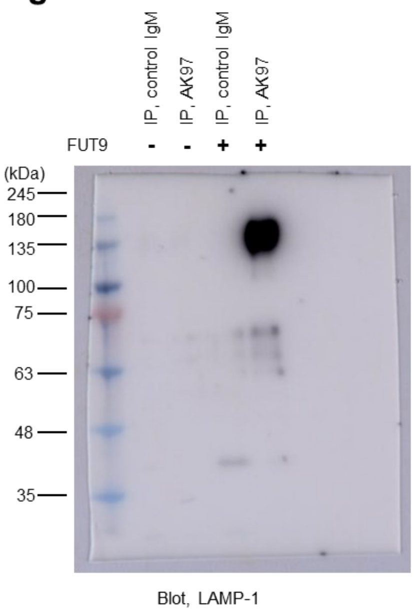

Supplementary Fig. 7. Unedited blot images presented in Fig. 1a-d

Fig. 1e

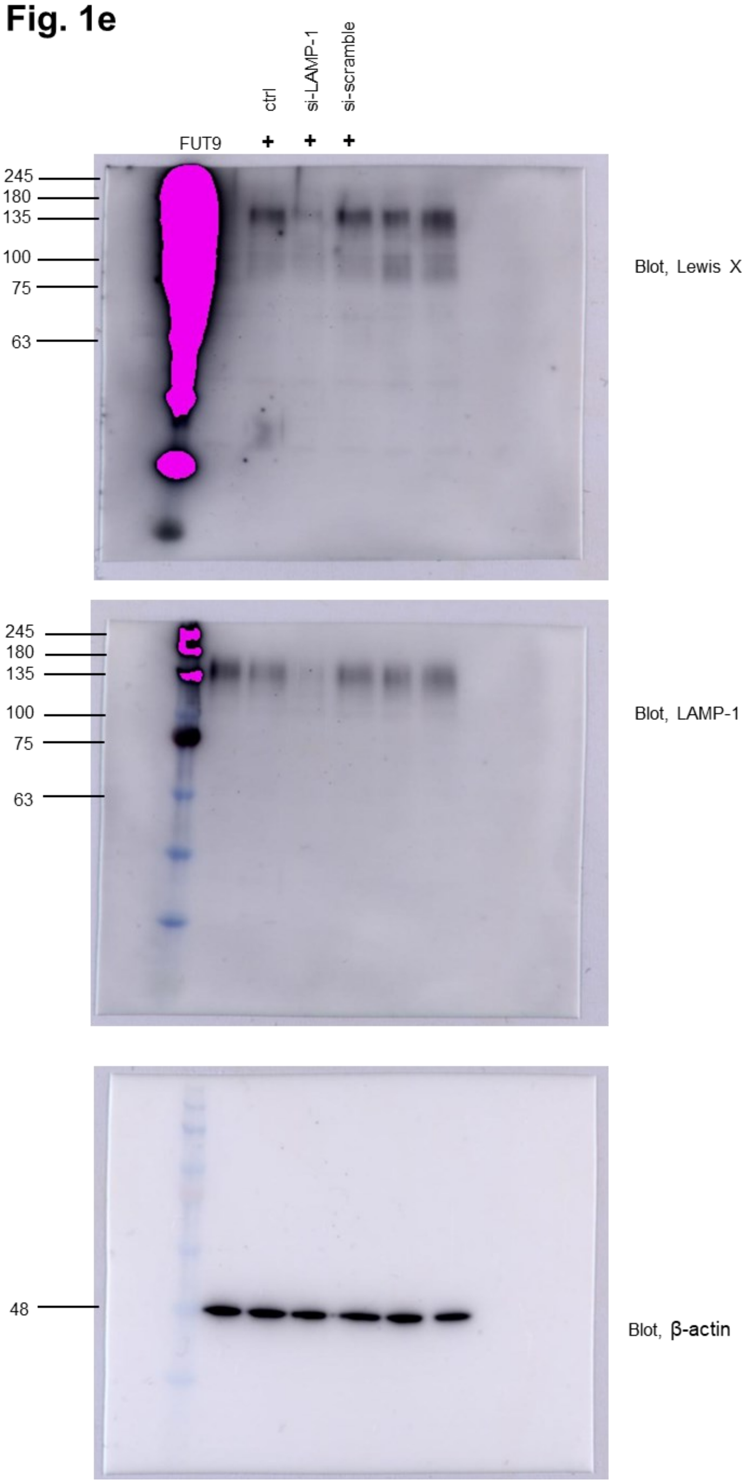

Supplementary Fig. 8. Unedited blot images presented in Fig. 1e

Fig. 3c

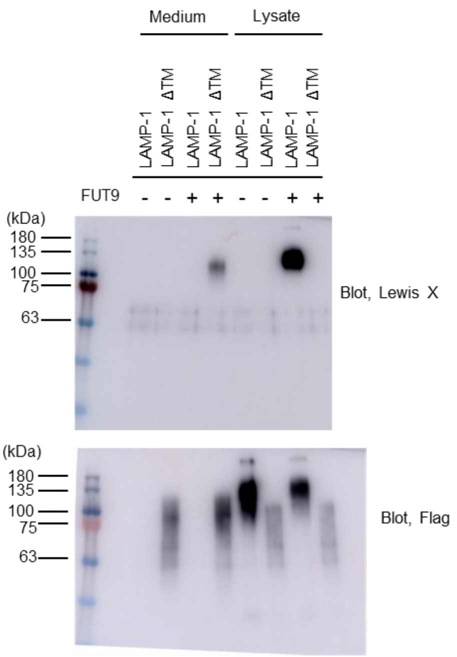

Fig. 3d

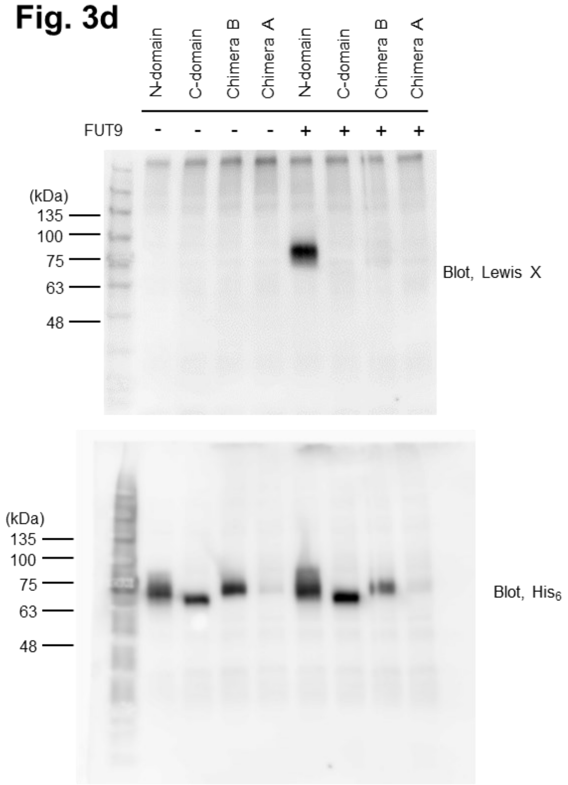

Fig. 3e

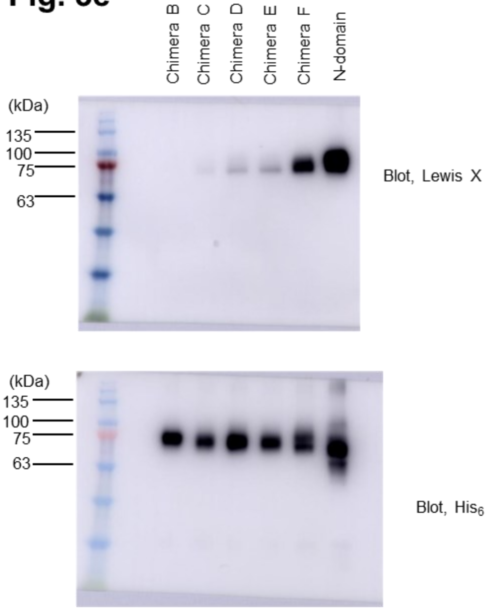

Fig. 3g

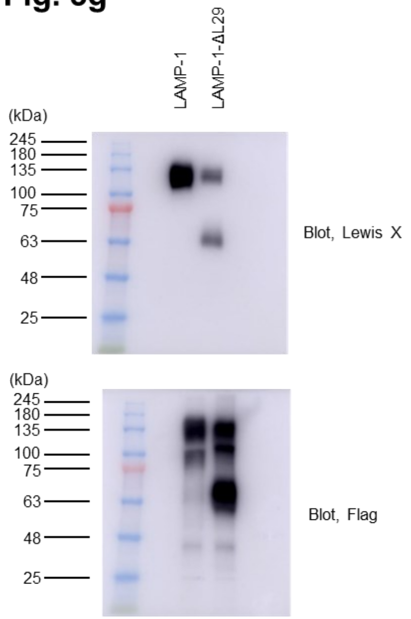

Supplementary Fig. 9. Unedited blot images presented in Figure 3c-e, g

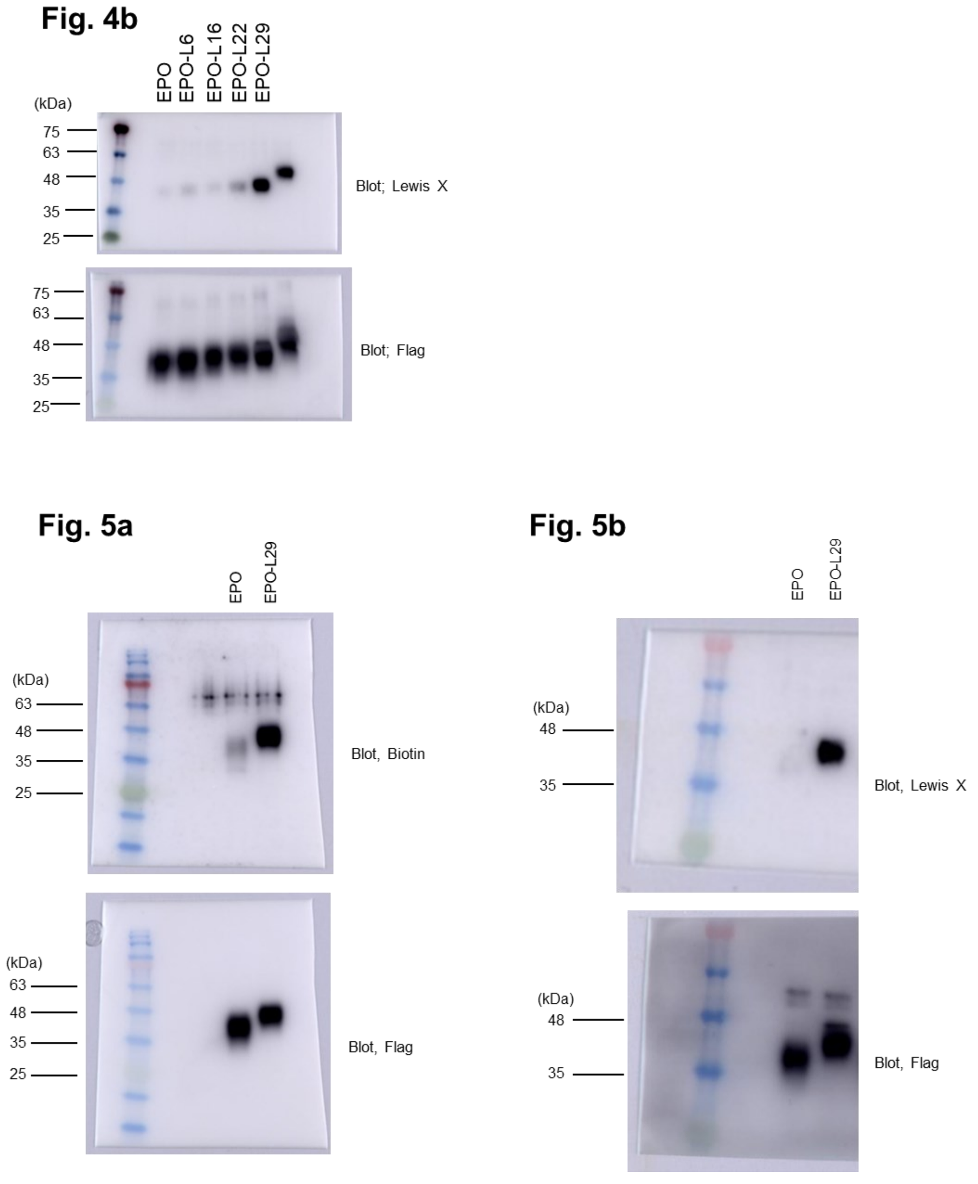

Supplementary Fig. 10. Unedited blot images presented in Fig. 4b, Fig. 5a, b

Supplementary Fig. 1

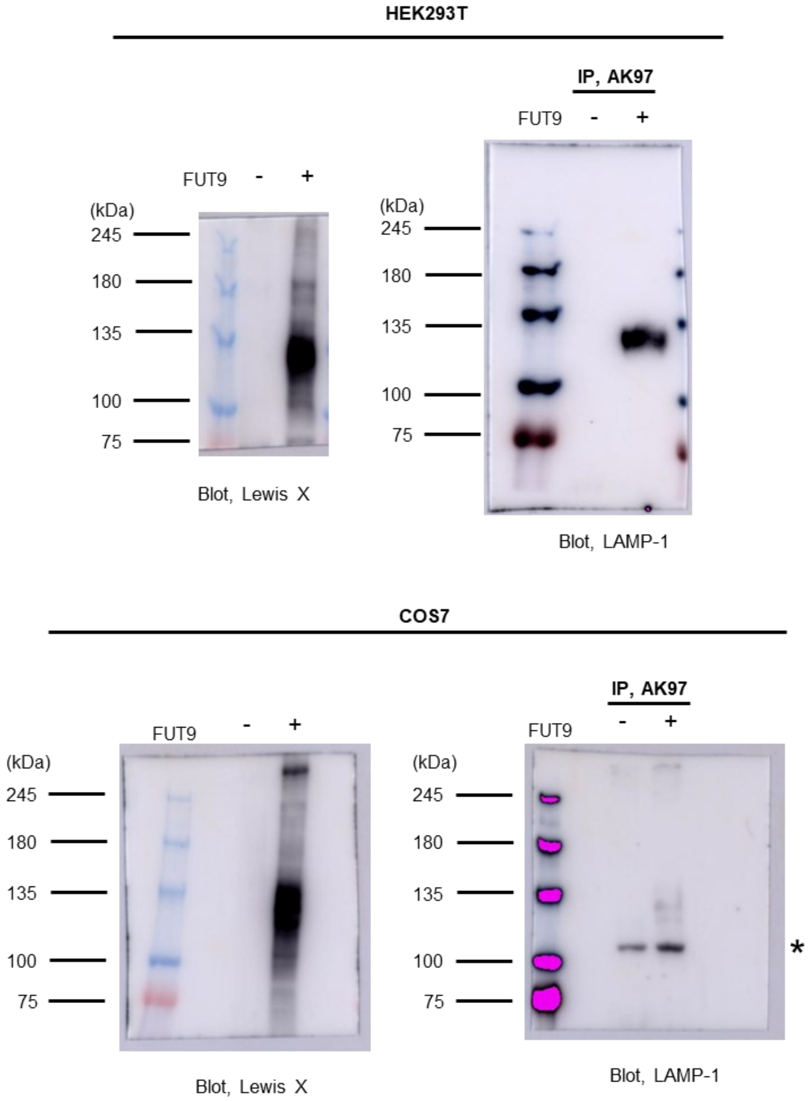

Supplementary Fig. 10. Unedited blot images presented in Supplementary Fig. 1

Supplementary Fig. 2

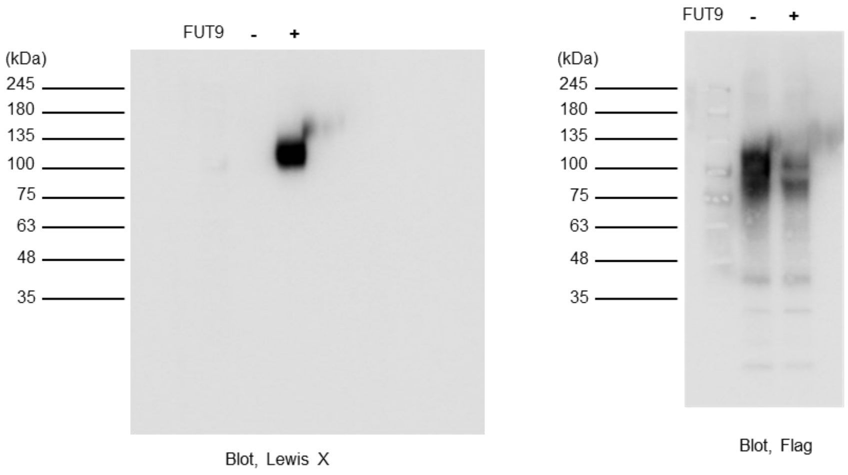

Supplementary Fig. 4

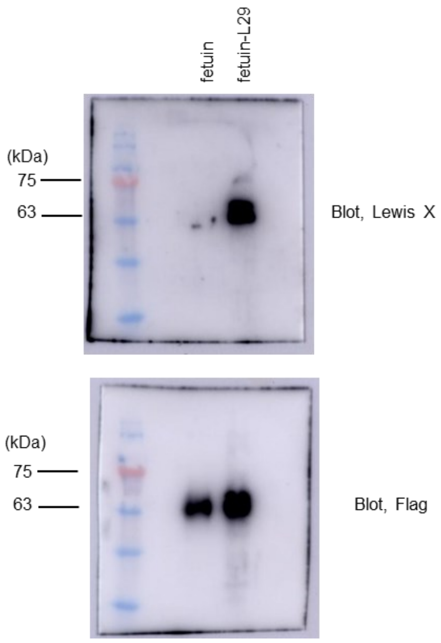

Supplementary Fig. 6

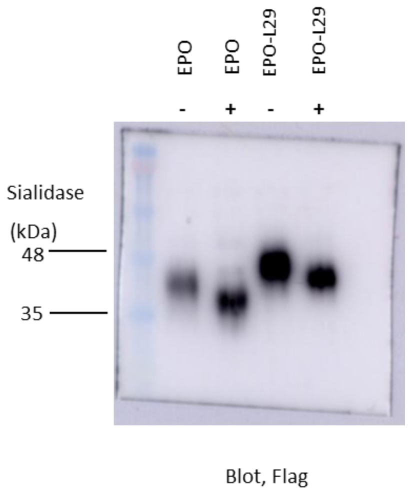

Supplementary Fig. 11. Unedited blot images presented in Supplementary Fig. 2, 4, 6
