# Supplementary material for: An embeddable molecular code for Lewis X modification through interaction with fucosyltransferase 9
Source: Commun Biol. 2022 Jul 13;5:676. doi: 10.1038/s42003-022-03616-1 (PMC9279290; doi:10.1038/s42003-022-03616-1)
Supplement: Supplementary file 3 — Description of Additional Supplementary Files [file 42003_2022_3616_MOESM3_ESM.pdf]

# Description of Additional Supplementary Files

**File name:** Supplementary Data 1
**Description:** The source data behind the graphs in the paper.

**File name:** Supplementary Data 2
**Description:** The Byonic search data.

**File name:** Supplementary Data 3
**Description:** The Byonic search data.
